# Compost from willow biomass (*Salix viminalis* L.) as a horticultural substrate alternative to peat in the production of vegetable transplants

**DOI:** 10.1038/s41598-022-22406-7

**Published:** 2022-10-21

**Authors:** Katarzyna Adamczewska-Sowińska, Józef Sowiński, Elżbieta Jamroz, Jakub Bekier

**Affiliations:** 1 Department of Horticulture, Wroclaw University of Environmental and Life Sciences, 50-375 Wrocław, Poland; 2 Institute of Agroecology and Crop Production, Wroclaw University of Environmental and Life Sciences, 50-375 Wrocław, Poland; 3 Institute of Soil Sciences, Plant Nutrition and Environmental Protection, Wroclaw University of Environmental and Life Sciences, 50-375 Wrocław, Poland

**Keywords:** Plant sciences, Environmental sciences

## Abstract

Willow (*Salix viminalis* L.) is a species well adapted to the environment conditions of central Europe. It is mainly cultivated for energy purposes as solid fuel. In this study, an evaluation of its suitability for other purposes was made using a 4-year old short rotation coppice (SRC) willow regrowth to produce chipped biomass which was composted. Four composting methods were used: without additives (WC), with the addition of nitrogen to narrow the C:N ratio (WN), with the addition of mycelium (WPG) and with the addition of mycelium and nitrogen (WPGN). A mixture of WC and WPGN composts was also prepared at 75:25% and 50:50% by volume. Composts, different proportion (25, 50 and 75%) of peat (SM) were evaluated for suitability as a substrate for tomato and cucumber transplant production. Tomato transplants produced in the medium were prepared from mixtures of willow composts (WPGN + WC(1) and WPGN + WC(2) and these mixtures with peat (WPGN + WC(1):SM and WPGN + WC(2):SM) were characterised as having the best parameters: plant height, lateral leaf span and number of leaves. Similarly, for cucumber transplants, better growth conditions than in peat substrate were obtained in the variant WPGN + WC(1) and WPGN + WC(1):SM. The addition of nitrogen to the composted biomass positively influenced the composting process. N concentration in the substrate was too high and toxic for the growth of tomato and cucumber transplants. At the end of the tomato and cucumber experiment, the nitrate content was 1510 and 2260 mg dm^−3^, respectively, in the WN substrate. Similarly, the high N–NO_3_^−^ content in the composted willow substrate with the addition of nitrogen and mycelium did not promote the growth of tomato and cucumber. Based on this research at least 25% of the mass of the peat can be replaced by different willow composts without having an adverse impact on seedling growth and with some of the willow compost mixtures this could be as high as 50%.

## Introduction

According to the Growing Media Europe, peat substrates are the primary means of production for the horticultural sector specialising in potted crops, with 750,000 employees and an estimated turnover of €60 billion^1^. Peat is the basic and most widely used substrate, whose use is estimated at 40 million m^3^ per year (representing nearly 68% of the volume of all substrates used in horticulture)^2,3^. Potentially much more can be utilised, but peat availability will reach limits in the foreseeable near future, primarily for environmental reasons^4–6^. Peat and peatlands have been classified as a resource of strategic global importance, and their exploitation should be sustainable and certified with further post-exploitation recultivation of the area^7^. Peatlands cover 2.86% of the earth's land area (4.36 million km^2^) and provide a number of beneficial ecosystem services such as C sequestration, water storage and water regulation, biodiversity conservation, environmental risk reduction, and recreational use^8,9^. The importance of peatlands, the need for their protection and restoration and their sustainable use was first identified in the Assessment on Peatlands, Biodiversity and Climate Change^6^. These area of lands play an important role in terms of water storage, thus having a beneficial effect on the environmental water cycle and reducing floods and droughts. According to Holden^10^, peatlands store as much as 10% of the available freshwater supply that feeds rivers. Drainage of peatlands and their use for agricultural production or exploitation (production of horticultural substrates), contributes to greenhouse gas emissions estimated at 1 billion tonnes CO_2_eq per year^11,12^.

Approximately 2000 km^2^ of peatlands are exploited for the production of horticultural substrates and 1 million km^2^ are used for agricultural or forestry production^13,14^. The vast majority (27 million m^3^) is used in European countries^4^, where peatlands account for about 25% of their total global area. Around 276,700 km^2^ of European peatlands, however, are considered degraded^15^. At the same time the value of horticultural substrates turnover in European countries accounts for more than 1.3 billion Euros, representing 11,000 employees^1^. Trends related to global economic and demographic development will contribute to the rapid growth of the substrate market. According to Blok et al.^3^, the use of substrates in the horticultural sector will increase by 445% over the next 30 years, while the production of substrates derived from wood and wood bark will increase by 1250 and 1000% respectively (compared to 2020)^1^. Taking into account the environmental aspects and the reduction of peat exploitation, the course of action for the coming years will be to replace peat or reduce its exploitation through the use of renewable materials^16^. One of the major approaches to reduce peat use relies on wood fibre and, for example, Hortfibre® technology^16–19^.

Replacing peat with other renewable materials is becoming a pressing issue of concern^16,20,21^. The alternative organic material used as a growing medium, must provide plant stabilization and also retain nutrients and water during plant growth^22^. Several natural peat substitutes have been used, such as coconut fibres, wood fibres, compost and many others^16,23–26^. Another way to produce alternative substrates is compost of lignin-cellulosic biomass obtained, for instance, from willow *Salix viminalis* L. grown in a 2–3 year cycle system as Short Rotation Coppice (SRC)^27,28^. Under aerobic decomposition, woody biomass (lignin-cellulosic) is converted into humus compounds (humic-fulvic complex)^29,30^. In European countries (Austria, Germany, UK, Italy), the share of composts in the total volume of substrates used is between 8 and 11%, and the total production of composts is estimated at 3 million m^3^^4^.

The suitability of substrates for the production of horticultural plants is determined by a number of parameters such as the ability to meet water and nutrient requirements, plant growth consistency and rooting ability of plants. For the production of safe substrates (without biological and chemical pollutants), it is crucial to ensure certain parameters of the composting process that provide final product suitable for plant growth^17,31^.

Composting is a part of the oxidative processes of biological and biochemical transformations that occur under strictly defined temperature, moisture and pH conditions, as well as C:N ratio. These factors affects the creation of suitable conditions in the composted material for microorganisms carrying out a cycle of organic matter transformation^32–34^. The most important aspect in the production of compost is to ensure the proper course of the organic matter transformation process, so that the product obtained does not pose a threat and brings substantial benefits as well as a positive impact on plant growth and development. Application of unstable and immature compost however inhibits germination and plant growth as indicated by many authors^35–38^. This is particularly important in relation to sanitary safety, reduction of greenhouse gas and odour emissions, stabilization of soil fertility parameters and prevention of excessive leaching of mineral compounds by small-molecule, mostly aliphatic organic substances^39,40^.

The use of compost and the adoption of organic farming practices is now one of the priority tasks of food production, driven by the growing demand for organic food by consumers^41^. Furthermore, composting is an environmentally friendly solution for the sustainable management of organic waste and one of the most reasonable methods for recycling mineral and organic substances^42^.

Mature compost is a very good source of stable organic matter containing very active humic substances. Due to the presence of nutrients that are readily available for plants, it can be successfully and efficiently used in agriculture. Stable humic substances present in compost influence directly and indirectly on plants. Results of the experiments showed that these active organic fractions can affect membrane permeability stimulating ions uptake or act as hormone-like substances, increasing root growth in plants^43^. Compost produced from biodegradable organic wastes/biomass is commonly described as a good amendment to improve structure, water-holding capacity, sorption properties as well as biological properties of soils and growing media^44–46^.

C:N ratio is a very significant parameter in ensuring proper course of the composting process. Most authors agree that 25–35 range for C:N is the most effective ratio for microorganism activity. Too high C:N ratio (over 35) can lead to prolonged organic matter decomposition duration, due to the lower activity of microorganisms while very low C:N ratio (below 20) increases the risk of nitrogen loss^47,48^.

Willow chips biomass is subject to slower and less effective humification, which results from a high C:N ratio, indicating the excess of degradable substrate for microbes^49^. Therefore one of the basic conditions for efficient initialization composting process is to narrow the C:N ratio by adding nitrogen to the composted biomass. Furthermore, willow biomass contains significant amounts of substances that slow down the mineralization processes like lignin^50^ as well as secondary metabolites of a high bioactivity e.g. phenolic compounds which are commonly known as composting inhibitors^51^. Composting process of willow biomass can be supported by use of additives that will break down lignin e.g. mycelium^52,53^.

Previous studies have shown that form of nitrogen used as an additive to composted biomass to improve the C:N ratio can significantly affect plant growth and development and in some cases adversely affects substrate quality^54^. Under highly saline conditions, plant growth was inhibited^55^. Mixing and confectioning materials with different parameters may be a way to provide optimal growth conditions for plants.

The aim of this study was to assess the usefulness of chopped willow composts produced by different methods and their mixtures as growing media for tomato and cucumber transplants. The study evaluated the usefulness of compost alone and compost as a peat substitute, in different proportions. A research hypothesis was adopted that peat used for the production of horticultural substrates could be replaced by nitrogen—enriched willow chip compost.

## Methods

### Experimental design and substrate preparing

Composting of chopped willow biomass was carried out in 2019 according to semi-dynamic open pile system^29,30,54,56–58^. For chopped willow production Junkkari HJ 4 M chopper was used. The chops size ranged from 4 to 12 mm. The composting process was varied by using additives to modify the process*:

- Willow composting without additives (WC),
- Willow composting with the addition of nitrogen (ammonium nitrate) to accelerate the biotransformation process (WN),
- Willow composting with the addition of fungi of the *Phanerochaetaceae* family (WPG), commercial product Pg Poszwald Eko,
- Willow composting with the addition of nitrogen and fungi belonging to the *Phanerochaetaceae* family (WPGN), commercial product Pg Poszwald Eko.

Prior to commencing the study, the matured compost was subjected to a grinding process using a Raffinatore Colibri COL/RR/2010 mill, POR Micucci System Srl—Italy. Particle size: 1 mm wide and 3–4 mm long. Ensuring adequate structure and particle size of biomass is the primary indicator for substrate evaluation. Substrate dry samples were sieved using an apparatus of Bakszajew sieve set at < 0.25, 0.25–0.50, 0.5–1.0, 1.0–3.0 and > 3.0 mm mesh diameter. Methods description performed at Adamczewska-Sowińska et al.^59^. Coarse compost fractions can hinder pot filling, provision of optimal moisture conditions, as well as root development and plant growth^60,61^.

Pot experiments were conducted under greenhouse conditions in two independent cycles to evaluate the suitability of willow composts for tomato and cucumber transplant production. Willow composts with and without different proportion of peat were used (Table 1). The control object was cultivation in peat substrate (SM), deacidified and supplied with nutrients. The share of substrate-forming components was determined in volume proportions.

**Table 1:** Horticulture media mixtures in % of volume.

Treatment code	Horticulture media code	Willow:peat proportion (%)	Media type and volume proportion (%)				
			WC	WN	WPG	WPGN	SM
1	WC	100:0	100	–	–	–	–
2	WC:SM	75:25	75	–	–	–	25
3	WC:SM	50:50	50	–	–	–	50
4	WC:SM	25:75	25	–	–	–	75
5	WN	100:0	–	100	–	–	–
6	WN:SM	75:25	–	75	–	–	25
7	WN:SM	50:50	–	50	–	–	50
8	WN:SM	25:75	–	25	–	–	75
9	WPG	100:0	–	–	100	–	–
10	WPG:SM	75:25	–	–	75	–	25
11	WPG:SM	50:50	–	–	50	–	50
12	WPG:SM	25:75	–	–	25	–	75
13	WPGN	100:0	–	–	–	100	–
14	WPGN:SM	75:25	–	–	–	75	25
15	WPGN:SM	50:50	–	–	–	50	50
16	WPGN:SM	25:75	–	–	–	25	75
17	WPGN + WC(1)	100:0	75	–	–	25	–
18	WPGN + WC(1):SM	75:25	56.25	–	–	18.75	25
19	WPGN + WC(1):SM	50:50	37.5	–	–	12.5	50
20	WPGN + WC(1):SM	25:75	18.75	–	–	6.25	75
21	WPGN + WC(2)	100:0	50	–	–	50	–
22	WPGN + WC(2):SM	75:25	37.5	–	–	37.5	25
23	WPGN + WC(2):SM	50:50	25.0	–	–	25.0	50
24	WPGN + WC(2):SM	25:75	12.5	–	–	12.5	75
25	SM	0:100	–	–	–	–	100

WC, willow compost; WC:SM, willow compost + sphagnum moss substrate; WN, willow compost with mineral nitrogen; WN:SM, willow compost with mineral nitrogen + sphagnum moss substrate; WPG, willow compost with *Phanerochaetaceae* decomposer; WPG:SM, willow compost with fungi decomposer + sphagnum moss substrate; WPGN, willow compost with *Phanerochaetaceae* decomposer and mineral nitrogen; WPGN:SM, willow compost with *Phanerochaetaceae* decomposer and mineral nitrogen + sphagnum moss substrate; WPGN + WC(1), willow compost mixtures in proportion 25:75%; WPGN + WC(1):SM, willow compost mixtures in proportion 25:75% + sphagnum moss substrate; WPGN + WC(2), willow compost mixtures in proportion 50:50%; WPGN + WC(2):SM, willow compost mixtures in proportion 50:50% + % + sphagnum moss substrate; SM, sphagnum moss substrate.

The resulting substrates and deacidified peat (pH 6.0) were thoroughly mixed with fertilizers so as to bring the nutrient content to the optimal value for tomato and cucumber transplants. These nutrient concentration were: 150 mg NH_4_NO_3_, 200 mg P, 250 mg K, 140 mg Mg and 1500 mg Ca in 1 dm^3^. Potassium monophosphate (1-potassium phosphate) containing 22.7% P and 28.2% K, ammonium nitrate (32% N), calcium-magnesium fertilizer containing 55% CaCO_3_, 42% MgCO_3_ and granulated fertilizer chalk 95–98% CaCO_3_ were applied. WN and WPGN medium were mixed without ammonium nitrate fertilizer.

The experiments were conducted in a greenhouse, on growing tables. The plants were flood irrigated. During the tomato transplants cultivation the temperature was maintained at 20–24 °C (until seedling emergence), and then at 18–20 °C. At night the temperature was lower by 3–5 °C. For cucumber, the temperature was maintained at 22–25 °C during the day and 18–20 °C during the night. Only natural light was used in the experiment (Table 1).

Seeds of tomato variety Awizo F1 (PlantiCo) were sown into boxes filled with 7 g m^−2^ of deacidified peat. Seedlings at the BBCH 10 development stage were planted into 248.6 cm^3^ pots filled with the prepared different substrates.

Seeds of cucumber variety Cezar F1 (Torseed) were sown directly into pots with the tested media.

There were 10 pots per substrate combination. The experiments were terminated when the plants reached the parameters allowing them to be planted in a permanent cultivation site. The experiment with tomato was terminated 4 weeks after seedling planting, while that with cucumber 3 weeks after sowing.

### Plant evaluation

Plant growth and development were evaluated during transplant production. Morphological measurements were made at weekly intervals determining the number of leaves, plant height and the largest leaf span. Plant condition was also evaluated according to the scale: (1) no plants (dying or phytotoxic substrate effect), (2) plant damaged, (3) plant wilted, (4) plant very weak (short, yellow turning purple plant, cotyledons not well developed), (5) plant weak (short plant, yellow cotyledons, leaves light green), (6) plant of medium condition (medium size plant, leaves light green), (7) plant of fairly good condition (medium size plant, leaves green), (8) plant of good condition (compacted plant, leaves green), (9) plant of very good condition (compacted plant, leaves intensive green). The experiment was completed with an additional evaluation of: weight of the aboveground parts of each plant, shoot diameter (1 cm above the soil surface), Soil Plant Analysis Development (SPAD), average weight of one leaf.

Leaf area was measured using a CID Bio-Science Portable Laser Leaf Area Meter CI-202 scanner by randomly selecting three leaves: largest, medium, and smallest from different plants, from each object. These leaves were also used to determine the average leaf unit weight. Based on the average area and number of leaves, leaf area per plant were calculated. Stocky plant index was calculated based on shoot length:shoot diameter ratio.

Upon termination of the experiments, chemical analysis of the media for N–NO_3_^−^, P, K, Ca, and Mg content was performed, pH and salinity were also determined. Chemical methods description performed at Adamczewska-Sowińska et al.^62^.

### Statistical analysis

The obtained results from morphological measurements of tomato and cucumber plants as well as quantitative and qualitative evaluation from particular measurement dates and before harvest were subjected to Anova/Manova analysis in Statistica software (version 13.1. StatSoft. Poland). All the analyses were performed at a significance level of P < 0.05 separately for each plant species. One- and two-factor analyses of variance were performed to evaluate the effect of horticultural substrates and different proportion of compost components in the substrate mixture on tomato and cucumber transplant characteristics. One-way analysis of variances were performed for horticultural substrate (WC, WN, WPG, WPGN, WPGN + WC(1), WPGN + WC(2), WC:SM, WN:SM, WPG:SM, WPGN:SM, WPGN + WC(1):SM, WPGN + WC(2):SM and SM) and compost proportion (25, 50, 75, 100). Two factors analyses were performed for the and interaction of compost:peat mixture substrate (6 treatments) with compost proportion (three treatments 25, 50, 75%).

The substrates type and the compost proportion of components in the mixture were the fixed effect of the statistical model, replicates the random effect of the model.

### Patents

Some of the results presented in this article were used in a patent filed on 28 June 2020 at the Polish Patent Office under No. P.435103. A legal procedure is currently underway.

## Results

### Size structure of substrate fractions

**Figure 1:**
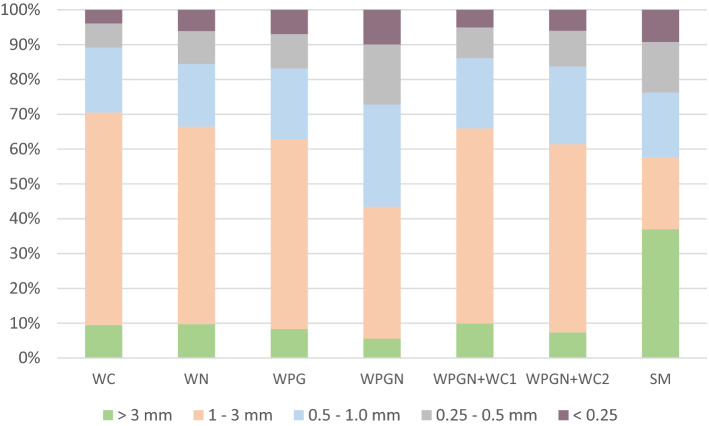
Horticulture media structure (% of biomass fraction). WC, willow compost; WN, willow compost with mineral nitrogen; WPG, willow compost with *Phanerochaetaceae* decom-poser; WPGN; willow compost with *Phanerochaetaceae* decomposer and mineral nitrogen; WPGN + WC(1), willow compost mixtures in proportion 25:75%; WPGN + WC(2), willow compost mixtures in proportion 50:50%; SM, sphagnum moss substrate.

The evaluation of the structure of the substrate obtained as a result of willow (WC) composting showed, in comparison with the peat substrate, almost four times lower content of the fraction of the largest particles and 2.4 times and twice lower content of fractions with diameter < 0.25 mm and 0.25–0.5 mm, respectively (Fig. 1). At the same time, this substrate contained three times more particles with a diameter of 1–3 mm. The addition of mineral nitrogen or fungi to willow chips during composting resulted in a decrease in the proportion of 1–3 mm particles and an increase in the content of fractions with a diameter of 0.25–0.5 mm and < 0.25 mm. The simultaneous application of nitrogen and fungi (WPGN) contributed to an even greater fineness of the substrate. The proportion of particles with diameter < 1 mm increased significantly, while the content of larger fractions decreased. In comparison with peat, this substrate contained 1.6 times more fractions of 0.5–1 mm diameter and 1.8 times more of 1–3 mm diameter, while at the same time the share of the largest particles was 6.6 times lower. The substrate created by mixing the WPGN compost with WC resulted in the formation of a product characterized by a smaller share of fractions 1–3 mm and a larger share of fractions < 1 mm compared to the WC basic compost.

### Evaluation of substrate suitability in tomato cultivation

**Figure 2:**
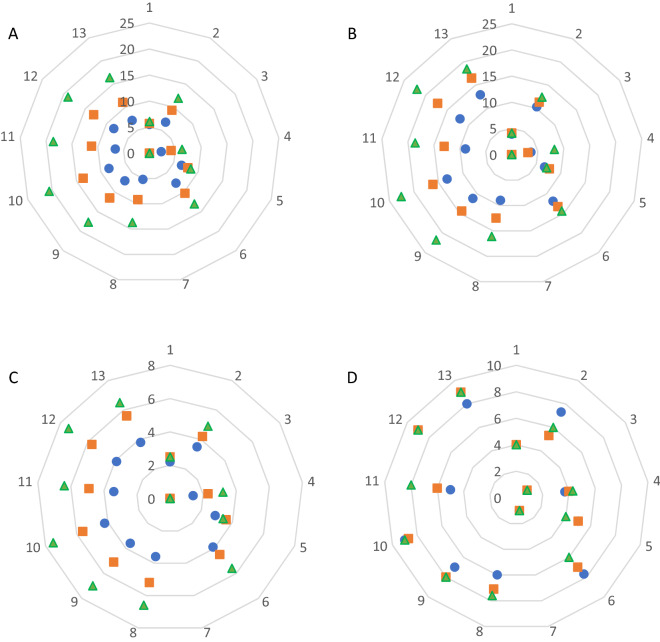
The effect of horticulture media on tomato plant parameters during vegetation. (**A**) Plant height (cm), (**B**) leaf lateral span (cm), (**C**) leaf number, (**D**) plant conditions (grade). Filled blue circle: two weeks. Filled orange square: three weeks. Filled green triangle: four weeks. 1-WC, 2-WC:SM, 3-WN, 4-WN:SM, 5-WPG, 6-WPG:SM, 7 WPGN, 8-WPGN:SM, 9-WPGN + WC(1), 10- WPGN + WC(1):SM, 11- WPGN + WC(2), 12-WPGN + WC(2):SM, 13-SM.

The study showed a strong, significant effect of substrate type on tomato plant growth (Table 2). On a homogeneous substrate prepared by composting willow chips with nitrogen or with the addition of nitrogen and fungi, the repotted tomato plants died. The addition of peat improved growth conditions, but until the end of cultivation transplant grown on substrate WN:SM and WPGN:SM was characterized by the worst parameters. The tallest plants were obtained on WPGN + WC(1) and WPGN + WC(2) mixtures with peat addition. The transplants grown on WPGN + WC(1) and WPGN + WC(2) or SM were also outstanding in height (Fig. 2A). It was observed that the increase in plant height between successive measurements in WC and WPG objects was 3.6–7% and 19.7–7.6%, and after mixing these substrates with peat 38.8–28% and 33.8–26.2%. In WPGN + WC(1) and WPGN + WC(2) and WPGN + WC(1):SM and WPGN + WC(2):SM, as well as SM, the growth rates ranged from 46.2 to 69.7%. Tomato cultivation in WN and WPGN media with peat addition resulted in plant height gains (between the first and second measurements) of 82.6% and 76.9%, respectively, and about 50% at the end of cultivation.

**Table 2:** Variance analysis for tomato plant depending on factors (significance verified by the Duncan test).

Measurement (weeks from seedling planting)	Treatment	Plant height	Leaf span	Number of leaf	Plant condition
Two	Media (M)	0.01	0.01	0.01	0.01
	Proportion (P)	< 0.001	0.01	< 0.001	0.01
	M × P	< 0.001	0.01	< 0.001	0.01
Three	Media (M)	0.01	0.01	0.01	0.01
	Proportion (P)	< 0.001	< 0.001	< 0.001	< 0.001
	M × P	0.01	0.01	0.01	0.01
Four	Media (M)	0.01	0.01	0.01	0.01
	Proportion (P)	< 0.001	< 0.001	< 0.001	< 0.001
	M × P	< 0.001	< 0.001	< 0.001	< 0.001

WC, willow compost; WN, willow compost with mineral nitrogen; WPG, willow compost with *Phanerochaetaceae* decomposer; WPGN, willow compost with *Phanerochaetaceae* decomposer and mineral nitrogen; WPGN + WC(1), willow compost mixtures in proportion 25:75%; WPGN + WC(2), willow compost mixtures in proportion 50:50%; SM, sphagnum moss substrate.

At the end of the transplant production period, the greatest plant span was observed for the WPGN + WC(1), WPGN + WC(1):SM, and WPGN + WC(2):SM (Fig. 2B). Tomato from the WPGN + WC(2) and SM group had a slightly smaller leaf span (16.2% on average) (Table 2).

On successive measurement dates, a systematic increase in plant height and spread was found under the influence of increasing peat content in the willow substrate. At the end of production, tomato transplants grown in mixtures with 25%, 50% and 75% peat content were on average 31.8%, 68.2%, 96.5% taller in comparison with those grown in homogeneous substrates and had 34.9%, 90.7% and 130.2% greater plant span. In the sites where the substrate was most conducive to transplant growth, after the addition of peat there was a 16.4% increase in tomato plant height (WPGN + WC(1):SM) compared to cultivation without peat, plant spread remained the same.

From the beginning of the growth period, plant condition was evaluated on a 9-point scale. Transplants grown in WPGN + WC(1):SM, WPGN + WC(2):SM and SM medium were rated highest (Fig. 2D). The condition of WPGN + WC(1) plants was fairly good to good, while WPGN + WC(2) plants were poor to medium at the beginning and good at the end of vegetation. In all cases, the addition of peat improved plant condition, which was most clearly visible when comparing WPGN and WPGN:SM plants. Increasing the proportion of peat in the substrate mixtures from 0 to 25%, 50% and 75% resulted in improved plant quality to those rated as medium, fairly good and good.

Tomato transplants ready for planting produced on WPGN + WC(1):SM and WPGN + WC(2):SM medium had the most leaves of the highest weight and unit area (Fig. 2C; Table 3). The stems of these plants had the largest diameter. The average entire plant weight and total leaf area per plant, as well as the Stocky index were among the highest and confirmed the very good condition and quality of this transplant. Similar quality was found when grown in peat substrate. In statistical terms, such parameters as weight and area of individual leaves were within the same homogeneous group, but their value was slightly lower than in the above described groups . Hence, the lower individual plant weight and total leaf area.

**Table 3:** The effects of horticulture media and components proportion on tomato transplant parameters.

Treatment	Transplant mass (g)	Steam diameter (mm)	Stocky plant index	SPAD	Leaf weight (g)	Leaf area (cm^2^)	Leaf area per plant (cm^2^)
**Plant growing media type**							
WC	0.4a	1.7b	38.5de	4.5a	0.2a	3.2a	8.0a
WC:SM	5.6b	3.7c	34.1cd	13.2ab	0.9a	24.6ab	146.7a
WN	0.0a	0.0a	0.0a	0.0a	0.0a	0.0a	0.0a
WN:SM	5.1b	2.1b	13.3b	27.9cd	1.3abc	38.9bc	227.5a
WPG	1.0a	2.4b	36.4de	6.4a	0.3a	7.4ab	25.5a
WPG:SM	5.9b	3.9cd	34.4cd	13.2ab	1.1ab	30.6ab	192.0a
WPGN	0.0a	0.0a	0.0a	0.0a	0.0a	0.0a	0.0a
WPGN:SM	12.2c	4.5cde	27.7c	39.6d	2.5cde	72.9d	511.1bc
WPNG + WC(1)	12.6c	4.7de	38.1de	21.2bc	2.3bcd	68.5cd	481.3b
WPNG + WC(1):SM	18.1d	5.2e	39.9de	23.5bc	3.7e	101.9d	772.0c
WPNG + WC(2)	11.8c	4.4cde	42.2e	25.6bcd	2.7de	83.3d	532.7bc
WPNG + WC(2):SM	16.8d	5.0e	38.5de	33.8cd	3.7e	100.9d	749.2bc
SM	14.4cd	5.0e	33.4cd	32.3cd	3.2de	88.8d	574.2bc
LSD (α = 0.05)	3.5	0.9	6.6	12.6	1.2	30.1	245.0
**Proportion of components (willow substrate:peat)**							
100:0	4.3a	2.2a	25.9	9.6a	0.9a	27.1a	174.6a
75:25	6.8b	3.0b	30.4	17.9a	1.4ab	41.8ab	289.8ab
50:50	9.9c	4.1c	30.1	28.6b	2.1b	58.2b	392.3b
25:75	15.0d	5.1d	33.4	29.1b	3.1c	84.9c	617.2c
LSD (α = 0.05)	2.3	0.6	r.n	8.5	0.9	24.3	191.2
**Media type × proportion of components interaction**							
LSD (α = 0.05)	2.9	0.7	7.0	9.6	0.9	27.1	174.6

Statistical analysis also showed similarity of some characteristics of transplants produced on WPGN + WC(1), WPGN + WC(2) and WPGN:SM in relation to the best quality plants. However, slightly lower individual leaf weight and area, smaller shoot diameter, and lower number of leaves per plant were found.

On the basis of the conducted analyses it was shown that chemical properties of the examined substrates depended on the components used in their production and their proportions (Table 4). After tomato transplants were grown: WN, WN:SM, WPGN, WPGN:SM substrates were characterized by a significantly lower reaction compared with the peat substrate. At the same time significantly higher salinity and nitrate content was found. The amount of P, K and Mg in WN and WPGN substrates was also among the highest. A tendency of decreasing salinity and macronutrient content in mixtures of these substrates with peat was observed. Significant change in salinity and P amount occurred in WN:SM, and phosphorus and magnesium in WPGN. The WPNG + WC(2) substrate was characterised by a lower reaction and Ca concentration, and a slightly higher content of other macronutrients in comparison with the peat substrate (Table 4).

**Table 4:** Horticulture media chemical composition—tomato.

Treatment	pH	mS/cm	Nitrate	P	K	Mg	Ca
			Mg dm^−3^				
**Plant growing media type**							
WC	7.0	0.2	2.5	107.0	238.0	60.0	312.0
WC:SM	6.7	0.6	4.9	75.0	191.0	55.7	831.7
WN	5.8	9.1	1510.0	158.0	275.0	134.0	570.0
WN:SM	6.0	5.9	1320.0	101.3	248.3	109.3	1026.7
WPG	7.2	0.5	1.0	107.0	275.0	54.0	370.0
WPG:SM	7.1	0.6	2.6	71.3	162.7	55.3	927.7
WPGN	5.5	5.2	1470.0	172.0	312.0	190.0	670.0
WPGN:SM	5.9	4.0	959.0	81.3	166.7	94.0	1160.0
WPNG + WC(1)	6.2	0.6	21.0	88.0	50.0	62.0	312.0
WPNG + WC(1):SM	6.4	0.5	9.1	66.3	39.0	63.0	967.7
WPNG + WC(2)	6.0	2.0	447.0	79.0	150.0	104.0	570.0
WPNG + WC(2):SM	6.3	0.9	141.7	54.0	38.3	73.7	1083.3
SM	6.6	1.2	45.0	58.0	52.0	76.0	2100.0
LSD (α = 0.05)	0.4	2.8	761.0	56.1	180.0	47.7	714.0
**Proportion of components (willow substrate:peat)**							
100:0	6.3	2.9	575.3	118.5	216.7	100.7	467.3
75:25	6.4	2.5	549.7	94.7	191.3	76.2	753.5
50:50	6.4	2.3	449.3	67.3	150.8	78.3	983.3
25:75	6.4	1.4	219.7	62.7	80.8	71.0	1261.7
LSD (α = 0.05)	n.s	n.s	n.s	30.7	n.s	n.s	185.0

The WC, WPG and WPGN:WC(1) substrates as well as their mixtures with peat exhibited salinity and N–NO_3_^−^, P and Mg contents at the same statistical levels as the peat substrate. However, there was a trend toward lower salinity (several times less) and nitrate content (approximately 2.1–45 times less) compared to the peat substrate.

Statistical analysis showed that an increase in the proportion of peat in the substrate caused a significant decrease in P and an increase in Ca. It also caused, not statistically confirmed, a tendency to a decrease in the salinity of the substrates and a decrease in the presence of the other macronutrients.

### Evaluation of the suitability of substrates in cucumber cultivation

Statistical analysis of the results of biometric measurements showed a significant effect of substrate type on cucumber transplant quality (Table 5).

**Table 5:** Variance analysis for cucumber plant depending on factors (significance verified by the Duncan test).

Measurement (weeks from sowing)	Treatment	Plant height	Leaf span	Number of leaf	Plant condition
One	Media (M)	0.01	0.01	0.01	n.i
	Proportion (P)	< 0.001	< 0.001	0.654	n.i
	M × P	< 0.001	0.01	< 0.001	n.i
Two	Media (M)	0.01	0.01	0.01	0.01
	Proportion (P)	< 0.001	< 0.001	< 0.001	< 0.001
	M × P	< 0.001	< 0.001	< 0.001	0.01
Three	Media (M)	0.01	0.01	0.01	0.01
	Proportion (P)	< 0.001	< 0.001	< 0.001	< 0.001
	M × P	< 0.001	< 0.001	0.01	0.01

n.i., no identificated.

**Figure 3:**
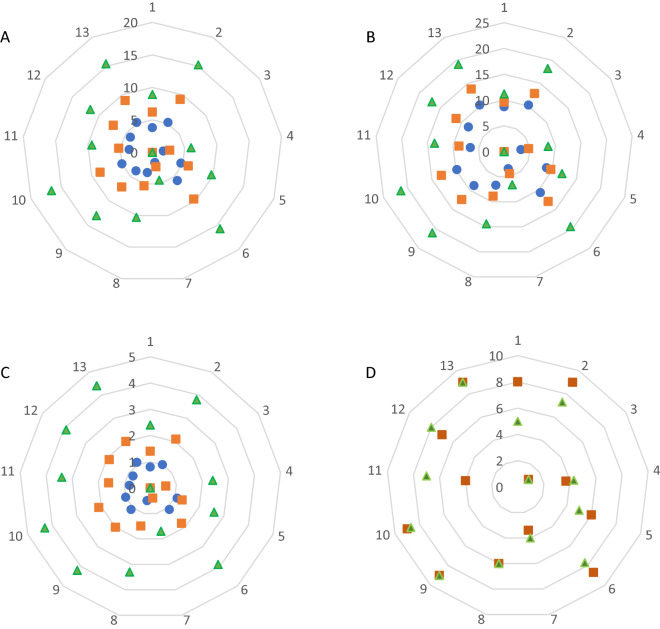
The effect of horticulture media on cucumber plant parameters during vegetation. (**A**) Plant height (cm), (**B**) leaf lateral span (cm), (**C**) leaf number, (**D**) plant conditions (grade). Filled blue circle: one weeks. Filled orange square: two weeks. Filled green triangle: three weeks. 1-WC, 2-WC:SM, 3-WN, 4-WN:SM, 5-WPG, 6-WPG:SM, 7 WPGN, 8-WPGN:SM, 9-WPGN + WC(1), 10- WPGN + WC(1):SM, 11- WPGN + WC(2), 12-WPGN + WC(2):SM, 13-SM.

It was found that, throughout the growth period, cucumber plants grown on WC:SM, WPG:SM, WPGN + WC(1):SM and SM medium were characterized by the greatest height (Fig. 3A). The substrate of composted willow with the addition of nitrogen or nitrogen and fungi caused the death of transplants (WN) or a considerable decline in their development (WN:SM, WPGN). A positive effect of peat addition to willow substrates—homogeneous ones and their mixtures—on plant height was observed throughout the whole cultivation period. Just before the termination of the experiment, cucumber grown on WC:SM and WPG:SM was on average 66.7% taller than on WC and WPG, and in the WPGN + WC(1):SM and WPGN + WC(2):SM objects by 24.6% taller than in WPGN + WC(1) and WPGN + WC(2). A 2.3-fold increase in height was observed at the WPGN:SM site compared to the control (Table 5).

The greatest leaf span of cucumber plants prior to termination was found in sites where WPGN + WC(1):SM and WPGN + WC(1), as well as SM and WPG:SM were used as substrates (Fig. 3B). Mixing WC and WPG substrates with peat resulted in approximately 62% increase in leaf span. It was proved that the addition of peat to willow substrates at 25%, 50% and 75% increased the height of cucumber plants by 40.8%, 69.7% and 86.8%, respectively, and the span by 30.8%, 50.5% and 82.2%.

Observations made after 14 and 21 days from the date of seed sowing recorded the greatest number of leaves cucumber plants growing on SM, WPGN + WC(1), and WPGN + WC(1):SM substrates, while slightly fewer leaves were recorded in WPGN + WC(2), WPGN + WC(2):SM, WPG:SM, and WC:SM objects (Fig. 3C). Cucumber plants grown in WPGN, WC, and WPG substrates produced 1.9–1.5 times fewer leaves than those growing in mixtures of these substrates with peat. An increase in the proportion of peat in the prepared media from 0 to 75% resulted in an increase in the number of leaves on plants on average from 2.4 to 4.2 leaves. The quality of plants determined on the adopted 9-degree scale significantly resulted from the method of substrate preparation for their cultivation. On the 14th and 21st day of growth the best quality was observed for transplants from SM and WPGN + WC(1), WPGN + WC(1):SM (Fig. 3D). On the 14th day of vegetation, plants from the WC:SM and WC and WPG:SM groups manifested similar quality (8–9). After another week of growth, the condition of these plants was rated lower, at 7.3–7.6 and even 5 (WC). The addition of peat and a gradual increase in its proportion in the basal substrates led to an improvement in plant condition from 5.2 to 7.5 on average.

Good quality of transplants grown on WPGN + WC(1), WPGN + WC(1):SM and SM, as well as WC:SM and WPG:SM substrates was confirmed by biometric measurements carried out at the end of their production. These plants were found to have on average higher unit weight (3.3-fold), shoot diameter (1.8-fold), weight per leaf (2.6-fold), area per leaf (2.7-fold), and leaf area per plant (3.6-fold) compared to the WC, WN, WN:SM, WPG, WPGN, WPGN:SM, WPGN + WC(2), WPGN + WC(2):SM (Table 6). The Stocky plant index in plants from most sites ranged from 21.2 to 26.5, with WPG:SM, WC:SM, WPGN + WC(1):SM, with SM transplants being the most stocky. Plants from WPGN and WPGN:SM sites were found to have the greenest leaves (SPAD) (Table 6).

**Table 6:** The effect of horticulture media and components proportion on cucumber transplant parameters.

Treatment	Transplant mass (g)	Steam diameter (g)	Stocky plant index	SPAD	Leaf weight (g)	Leaf area (cm^2^)	Leaf area per plant (cm^2^)
**Plant growing media type**							
WC	3.9ab	4.5c	20.1bcd	11.7ab	2.5ab	87.2bc	215.1ab
WC:SM	13.3de	6.4ef	25.9d	17.5bc	6.6 cd	225.1ef	912.0 cd
WN	0.0a	0.0a	0.0a	0.0a	0.0a	0.0a	0.0a
WN:SM	3.5ab	2.7b	13.7b	35.3cde	2.1ab	68.1abc	244.2ab
WPG	3.0ab	4.2c	23.3d	12.2ab	2.0ab	54.5ab	142.8ab
WPG:SM	13.3de	6.0def	26.5d	22.9bcd	6.4cd	212.0ef	831.2cd
WPGN	3.3ab	2.9b	15.0bc	41.5e	2.8b	61.2abc	112.2a
WPGN:SM	6.9bc	4.9cd	21.4cd	39.6de	3.3b	112.4bc	394.3ab
WPNG + WC(1)	12.6de	6.5f.	20.0bcd	27.1bcde	6.4cd	195.4def	824.3cd
WPNG + WC(1):SM	17.3e	6.6f.	25.1d	28.2bcde	8.1d	268.3f.	1158.1d
WPNG + WC(2)	4.9bc	4.4c	21.2cd	25.9bcde	3.9bc	122.6bcd	413.2ab
WPNG + WC(2):SM	9.3cd	5.2cde	22.6d	35.6cde	4.2bc	142.7cde	553.6bc
SM	15.2e	6.3ef	24.6d	30.2cde	6.2cd	204.1def	907.9cd
LSD (α = 0.05)	4.3	1.2	6.1	15.7	2.4	76.5	371.0
**Proportion of components (willow substrate:peat)**							
100:0	4.6a	3.7a	16.6a	19.7a	2.9a	86.8a	284.6a
75:25	8.2b	4.5ab	20.6ab	21.6a	4.4ab	140.6ab	530.8ab
50:50	9.9b	5.3bc	23.8b	32.8b	4.9b	168.0bc	641.9cd
25:75	13.7c	6.1c	23.2b	35.2b	6.1c	205.7c	874.0d
LSD (α = 0.05)	2.9	0.9	4.0	9.1	1.7	55.9	256.4
**Media type × proportion of components interaction**							
LSD (α = 0.05)	4.2	0.9	6.0	8.6	2.4	67.5	295.0

Substrate chemical analyses performed after the experiment and statistical analysis of the results showed that the chemical composition depended on the substrate type (Table 7). The composted willow substrate (WC, WC:SM) exhibited pH, salinity, and N-NOˉ_3_, K, and Mg at the same statistical levels as the peat substrate. However, there was a trend toward lower salinity (several times lower) and nitrate content (approximately 25 and 16 times lower) compared to the peat substrate. The phosphorus content, on the other hand, was on average twice higher here. In WN and WN:SM media the pH was determined at pH 5.8, while salinity was 4.5–3.5 times higher than in SM. In contrast, salinity was 4.5 to 3.5 times greater than in the SM and was due to a significantly higher content of N-NOˉ_3_ and other macronutrients. It was observed that the addition of peat to the WN basic compost resulted in a decrease in the final content of nitrate, P, K and Mg and, as a result, a decrease in the salinity of the WN:SM substrate (Table 7).

**Table 7:** Horticulture media chemical composition—cucumber.

Treatment	pH	mS/cm	Nitrate	P	K	Mg	Ca
			mg dm^−3^				
**Plant growing media type**							
WC	6.6	0.3	2.5	112.0	75.0	54.0	338.0
WC:SM	6.8	0.6	3.9	132.3	121.0	58.7	685.0
WN	5.8	5.9	2260.0	155.0	405.0	250.0	825.0
WN:SM	5.8	4.6	1295.7	112.0	279.3	154.0	1125.0
WPG	7.2	0.3	3.4	84.0	100.0	48.0	575.0
WPG:SM	7.1	0.5	5.2	81.7	54.0	57.3	662.7
WPGN	4.9	3.8	856.0	138.0	135.0	192.0	638.0
WPGN:SM	5.4	3.4	623.3	104.7	128.3	99.0	793.3
WPNG + WC(1)	6.6	0.3	3.8	122.0	75.0	54.0	650.0
WPNG + WC(1):SM	6.8	0.5	11.1	70.3	27.7	42.7	952.0
WPNG + WC(2)	5.9	1.2	160.0	83.0	25.0	84.0	425.0
WPNG + WC(2):SM	5.9	1.8	243.3	75.0	57.3	63.3	675.0
SM	6.3	1.3	64.0	61.0	78.0	59.0	1045.0
LSD (α = 0.05)	0.5	1.4	1134.0	45.6	205.0	76.4	n.s
**Proportion of components (willow substrate:peat)**							
100:0	6.2	2.0	547.6	115.7	135.8	113.7	575.2
75:25	6.3	1.9	558.3	110.8	155.3	91.0	668.8
50:50	6.3	2.1	285.7	93.0	108.0	78.0	996.7
25:75	6.3	1.7	247.4	84.2	70.5	68.5	781.0
LSD (α = 0.05)	n.s	n.s	n.s	n.s	n.s	n.s	n.s

Compared to peat substrate, lower pH and higher (2.9- and 2.6-fold) salinity after cucumber cultivation were observed in WPGN and WPGN:SM substrates. On average, the WPGN medium exhibited 13.4-fold greater N-NOˉ_3_, 3.3-fold greater P, 1.7-fold greater K, and 3.3-fold greater Mg. Correspondingly, the WPGN:SM combination resulted in 27.2% less nitrate, 24.1% less P and 48.4% less Mg concentration. The pH, salinity, nitrate, K and Mg contents of WPGN + WC(1), WPGN + WC(1):SM and WPGN + WC(2), WPGN + WC(2):SM substrates were at the same level of significance as those of SM. However, when comparing the substrates based on the WPGN and WC compost mixtures, it was found that the higher proportion of WC (75%) provided a higher pH and a trend toward lower salinity with significantly less N–NO_3_^−^.

There was no significant effect of the proportion of SM in the mixtures, but it was noted that increasing the amount of this component from 0 to 75% resulted in a decrease in the salinity of the substrate and in the content of nitrates, P, K and Mg.

## Discussion

One of the most important criteria for evaluating a peat substitute is its particle size. It is recommended, before use, to grind it to a diameter below 10 mm^28^. In the conducted studies, the majority of prepared composts and compost mixtures showed a dominant share of fractions with a diameter of 1–3 mm (from 54%—WPGN + WC2 to 61%—WC). A higher proportion of fractions < 1 mm amounting to 56.5% was found after the addition of mycelium and nitrogen (WPGN) in the composting process. The addition of nitrogen decreased the C:N ratio and increased the activity of microorganisms decomposing cellulose-lignin biomass^47^. Additionally, during the composting process, microbial activity was increased with the addition of mycelium (WPGN). The composting of willow without additives had the smallest effect on the proportion of fractions < 1 mm in diameter (29.5%). Secondary clumping occurred in the peat and the proportion of fractions > 3 mm was the highest (37.0%). In the study by Zawadzinska et al.^63^, the share of fractions below 1 mm ranged from 20% in compost to 29–42% in substrates with compost mixtures. A larger share of finer fractions favourably affects the increase in water capacity of the substrate^64^. Conditions for optimum plant growth are provided by particles with a granulation of 0.25–2.0 mm ensuring the availability of water and air^44,65^. In the tests carried out, the WPGN substrate was distinguished in terms of the proportion of finest fractions. This did not transfer to the growth of tomato and cucumber seedlings in this substrate.

A horticultural substrate consisting of a peat mixture with the addition of wood fibre compost provided a high yield of fruit of comparable or higher nutritional value than in peat substrates^63^. According to the authors, the higher nutrient content and their availability to the tomato plants and the larger pore space had a beneficial effect on the yield of this species and the quality of its fruit. In our study, better parameters of tomato transplants (plant height, leaves span and number of leaves) were obtained in substrates prepared from willow compost than in homogeneous peat substrates (compost mixtures WPGN + WC(1) and WPGN + WC(2) and compost-peat mixtures WPGN + WC(1):SM and WPGN + WC(2):SM). In the evaluation of the effect of substrates on morphological characteristics of cucumber transplants, better parameters than in peat substrates were obtained in the WPGN + WC(1) and WPGN + WC(1):SM variants. The weight of tomato transplants was 16.7–25.7% higher in the cultivation on WPGN + WC(1):SM and WPGN + WC(2):SM mixed media than that obtained on peat substrate. In the production of cucumber transplants, only in the WPNG + WC(1):SM variant a 13.8% higher weight was obtained than on peat substrate. The mixture of WPGN and WC composts ensured the best availability of nutrients to tomato and cucumber seedlings, while at the same time the appropriate fractional composition of the substrate positively influenced the development of the root system. This resulted in better seedling growth, their morphological characteristics and weight. Zwadzińska et al.^63^ and Verma et al.^66^ point out that the obtained plant weight depends on the availability of nutrients and the rate of their uptake from the substrate. The SPAD value indicated good seedling nutrition in the SM, WPNG + WC(1), WPNG + WC(1):SM, WPNG + WC(2), WPNG + WC(2):SM variants.

Willow biomass has a high total organic carbon (TOC) content of 478.9 g kg^−1^^30^. Although the use of composting to process this type of substrate is accepted, the most important problem associated with this type of substrates are the secondary metabolites of a high bioactivity e.g. phenolic compounds which are commonly known as composting inhibitors^51^. Modification of the composting by the addition of nitrogen and mycelium^30,54^ positively affected the substrate quality parameters after the experiment. Substrates without nitrogen addition (WC and WPG) were characterized by low nitrate content (from 1.0 to 4.9 mg per 1 dm^3^ after tomato cultivation and from 2.5 to 5.2 mg per 1 dm^3^ after cucumber cultivation). This was confirmed by the very low SPAD values. In media obtained from compost, which was produced under conditions of artificially increased nitrogen availability, nitrate content was very high, reaching a maximum of 1510 and 2260 mg per 1 dm^3^ in the WN variant, following tomato and cucumber cultivation, respectively. The amount of nitrogen added should be based not only on the optimal C:N ratio for composting, but also on what the target nitrogen content of the resulting substrate and its salinity should be. In subsequent trials, the amount of nitrogen added during the composting process should be reduced by at least one third or the source of nitrogen should be changed to organic biomass, for example.

The salinity of the substrate also results from the nitrate anion (NO_3_^−^) content^67^. The addition of nitrogen to accelerate the composting process and reduce the C:N ratio contributed to the deterioration of substrate parameters with salinity above 4 mS cm^−1^ (from 4.6—substrate from WN:SM cucumber cultivation to 9.1 mS cm^−1^—WN substrate sampled after tomato cultivation). Gondek et al.^67^ report that at salinity above 4 mS cm^−1^ soils are classified as saline. In soilless media, cucumber cultivation is recommended when salinity is below 2.5 mS cm^−1^^68^. Above this value, its growth is inhibited by 13% per one unit increase in salinity. In studies on tomato, a significant decrease in yield was recorded when grown under salinity conditions above 3.5 mS cm^−1^^69^. In our study, no cucumber emergence and growth was found in the WN variant, while tomato was found in the WN and WPGN variants.

The ratio of plant stem length to diameter^70^ or weight to diameter^71^ determines the strength of the transplant, and as this ratio decreases the transplant is stronger and more compact. In tomato, the ratio of height to stem diameter at the base varied extensively from 13.3 to 42.2. In a study by Uçan and Ugur^72^, the ratio depended on the developmental stage of the tomato and at 50 days after sowing, it was in a similar range (from 11.4 to 31.0), reaching a maximum value on the 81st day of vegetation—63.6. In the study conducted by Díaz-Pérez and Camacho-Ferre^73^, the ratio of plant height to diameter was within a narrower range and ranged from 38.9 to 50.7. At the lower range was the value for tomato transplants produced on peat substrate—43.8. Similarly, in our study, tomato plants from peat substrate had an index of 33.4. However, there was a tendency for it to increase from 25.9 in 100% willow compost substrates to 33.4 when the ratio of willow to peat in the substrate was 25:75%. A more compact transplant is more resistant to transplanting^73^. Plants with a high stocky index, when pulled out are more sensitive to transplanting stress. The tomato seedling was the most compact with the peat substrate. In cucumber, the best seedlings were obtained in WPNG + WC(1), WPNG + WC(2) and WPNG + WC(2):SM medium.

In cucumber, the ratio of height to stem diameter at the base ranged from 13.7 to 25.1. The same relationship was found and the index increased significantly from 16.6 to 23.2 when the proportion of peat in the substrate was increased from 0 to 75%.

## Conclusion

The reaction of tomato and cucumber in the initial growth period to the applied substrates was similar. At the same time, the obtained results indicate the need for further modification of the willow biomass composting and confectioning process. The addition of nitrogen during composting is necessary to initiate and sustain the process of biotransformation of cellulose-lignin compounds into humic compounds. Therefore, compost mixtures WPGN + WC(1) and WPGN + WC(2) provided the best production results comparable to those obtained from tomato and cucumber transplant production in peat substrate.

Based on the results obtained from the conducted studies, it can be recommended to reduce the proportion of peat in the substrate by 50%, and in some variants by 75%. This measure will significantly reduce the exploitation of peat from environmentally valuable communities.

### Permission to collect species used in experiment

In the conducted research, cultivated crop and commercial fungi were used. According to the national regulations, the use of these species for experimental purposes does not require any special permit. Our study complies with relevant institutional, national, and international guidelines and legislation.

## Supplementary Information

Supplementary Information.

## Data Availability

Authors can confirm that all relevant data are included in the given article.
