# Supplementary material for: Compost from willow biomass (Salix viminalis L.) as a horticultural substrate alternative to peat in the production of vegetable transplants
Source: Sci Rep. 2022 Oct 21;12:17617. doi: 10.1038/s41598-022-22406-7 (PMC9587216; doi:10.1038/s41598-022-22406-7)
Supplement: Supplementary file 1 — Supplementary Information. [file 41598_2022_22406_MOESM1_ESM.docx]

Table S1. The interaction of horticulture media and willow substrate:peat proportion on tomato plants parameters. Two weeks from seedling planting

| Horticulture  media | Willow substrate:peat proportion | Plant height  (cm) | Leaf span  (cm) | Number of leaf | Plant conditions |
| --- | --- | --- | --- | --- | --- |
|  | 75:25 | 5.0 | 5.5 | 2.6 | 5.0 |
| WC:SM | 50:50 | 7.2 | 11.7 | 3.9 | 8.0 |
|  | 25:75 | 8.0 | 13.6 | 4.1 | 8.9 |
|  | 75:25 | 0.0 | 0.0 | 0.0 | 1.0 |
| WN:SM | 50:50 | 1.7 | 2.1 | 1.0 | 2.0 |
|  | 25:75 | 5.2 | 9.1 | 3.3 | 8.0 |
|  | 75:25 | 7.0 | 9.4 | 3.4 | 6.0 |
| WPG:SM | 50:50 | 8.0 | 12.5 | 3.9 | 8.0 |
|  | 25:75 | 8.0 | 13.8 | 4.3 | 9.0 |
|  | 75:25 | 3.1 | 4.6 | 2.4 | 4.0 |
| WPGN:SM | 50:50 | 5.3 | 9.5 | 4.0 | 5.0 |
|  | 25:75 | 7.1 | 12.8 | 4.4 | 9.0 |
|  | 75:25 | 8.6 | 12.5 | 4.1 | 9.0 |
| WPNG+WC(1):SM | 50:50 | 8.3 | 14.1 | 4.4 | 9.0 |
|  | 25:75 | 7.9 | 13.0 | 4.1 | 9.0 |
|  | 75:25 | 8.5 | 12.0 | 4.0 | 9.0 |
| WPNG+WC(2):SM | 50:50 | 8.0 | 11.4 | 3.9 | 9.0 |
|  | 25:75 | 8.5 | 12.7 | 3.9 | 9.0 |
| **LSD (α=0.05)** | | **1.2** | **1.7** | **0.7** | **0.1** |

Table S2. The interaction of horticulture media and willow substrate:peat proportion on tomato plants parameters. Three weeks from seedling planting

| Horticulture  media | Willow substrate:peat proportion | Plant height  (cm) | Leaf span  (cm) | Number of leaf | Plant conditions |
| --- | --- | --- | --- | --- | --- |
|  | 75:25 | 6.3 | 5.1 | 2.8 | 4.0 |
| WC:SM | 50:50 | 9.7 | 12.4 | 4.5 | 5.0 |
|  | 25:75 | 11.9 | 16.3 | 5.4 | 7.0 |
|  | 75:25 | 0.0 | 0.0 | 0.0 | 1.0 |
| WN:SM | 50:50 | 3.0 | 4.2 | 2.0 | 3.0 |
|  | 25:75 | 9.7 | 5.1 | 5.0 | 8.0 |
|  | 75:25 | 8.4 | 9.4 | 3.4 | 5.0 |
| WPG:SM | 50:50 | 10.8 | 14.0 | 4.7 | 7.0 |
|  | 25:75 | 11.8 | 16.5 | 5.5 | 9.0 |
|  | 75:25 | 5.6 | 8.3 | 3.7 | 4.2 |
| WPGN:SM | 50:50 | 10.0 | 13.9 | 5.8 | 8.0 |
|  | 25:75 | 11.9 | 15.2 | 6.2 | 9.0 |
|  | 75:25 | 13.6 | 15.4 | 5.5 | 9.0 |
| WPNG+WC(1):SM | 50:50 | 14.1 | 16.8 | 5.7 | 9.0 |
|  | 25:75 | 13.2 | 16.0 | 5.7 | 8.0 |
|  | 75:25 | 13.1 | 16.5 | 5.7 | 9.0 |
| WPNG+WC(2):SM | 50:50 | 12.9 | 17.6 | 5.6 | 9.0 |
|  | 25:75 | 12.9 | 17.4 | 5.9 | 9.0 |
| **LSD (α=0.05)** | | **1.7** | **2.3** | **0.8** | **0.6** |

Table S3. The interaction of horticulture media and willow substrate:peat proportion on tomato plants parameters. Four weeks from seedling planting

| Horticulture  media | Willow substrate:peat proportion | Plant height  (cm) | Leaf span  (cm) | Number of leaf | Plant conditions |
| --- | --- | --- | --- | --- | --- |
|  | 75:25 | 7.5 | 5.6 | 3.1 | 4.0 |
| WC:SM | 50:50 | 12.9 | 14.5 | 5.2 | 6.0 |
|  | 25:75 | 15.4 | 17.0 | 6.5 | 8.0 |
|  | 75:25 | 0.0 | 0.0 | 0.0 | 1.0 |
| WN:SM | 50:50 | 5.3 | 6.0 | 2.7 | 4.0 |
|  | 25:75 | 13.5 | 18.7 | 7.0 | 8.0 |
|  | 75:25 | 9.8 | 9.3 | 4.2 | 5.0 |
| WPG:SM | 50:50 | 14.8 | 16.4 | 5.7 | 6.0 |
|  | 25:75 | 14.3 | 17.4 | 6.8 | 7.0 |
|  | 75:25 | 9.7 | 11.7 | 5.0 | 6.6 |
| WPGN:SM | 50:50 | 13.8 | 16.4 | 7.0 | 7.3 |
|  | 25:75 | 17.6 | 20.3 | 7.8 | 9.0 |
|  | 75:25 | 20.6 | 22.0 | 7.7 | 9.0 |
| WPNG+WC(1):SM | 50:50 | 20.2 | 22.1 | 7.6 | 9.0 |
|  | 25:75 | 21.0 | 23.7 | 7.3 | 9.0 |
|  | 75:25 | 19.7 | 21.0 | 7.5 | 9.0 |
| WPNG+WC(2):SM | 50:50 | 18.8 | 23.2 | 7.4 | 9.0 |
|  | 25:75 | 18.6 | 21.9 | 7.4 | 9.0 |
| **LSD (α=0.05)** | | 2.6 | 3.2 | 1.2 | 1.0 |

Table S4. The interaction of horticulture media and willow substrate:peat proportion on tomato on tomato transplant parameters

| Horticulture  media | Willow substrate:peat proportion | Transplant mass (g) | Steam diameter (mm) | Stocky plant index | SPAD | Leaf weight (g) | Leaf area (cm^2^) | Leaf area per plant (cm^2^) |
| --- | --- | --- | --- | --- | --- | --- | --- | --- |
|  | 75:25 | 0.7 | 2.0 | 39.1 | 6.0 | 0.2 | 5.2 | 16.9 |
| WC:SM | 50:50 | 4.7 | 4.0 | 32.7 | 14.9 | 0.7 | 17.9 | 93.7 |
|  | 25:75 | 11.3 | 5.1 | 30.6 | 18.6 | 1.9 | 50.7 | 329.4 |
|  | 75:25 | 0.0 | 0.0 | 0.0 | 0.0 | 0.0 | 0.0 | 0.0 |
| WN:SM | 50:50 | 1.5 | 1.4 | 11.3 | 43.6 | 0.9 | 31.4 | 80.4 |
|  | 25:75 | 13.7 | 4.8 | 28.5 | 40.0 | 2.9 | 85.3 | 602.1 |
|  | 75:25 | 1.6 | 2.7 | 37.5 | 7.5 | 0.4 | 9.5 | 40.6 |
| WPG:SM | 50:50 | 5.7 | 4.3 | 34.6 | 13.4 | 1.0 | 26.8 | 154.6 |
|  | 25:75 | 10.3 | 4.7 | 30.9 | 18.7 | 2.0 | 55.6 | 380.9 |
|  | 75:25 | 5.8 | 3.4 | 23.4 | 40.8 | 1.3 | 46.9 | 239.6 |
| WPGN:SM | 50:50 | 12.3 | 4.6 | 27.2 | 42.6 | 2.4 | 68.7 | 493.3 |
|  | 25:75 | 18.4 | 5.6 | 32.5 | 35.3 | 3.9 | 103.0 | 800.5 |
|  | 75:25 | 17.0 | 5.0 | 41.6 | 20.6 | 3.3 | 95.2 | 738.1 |
| WPNG+WC(1):SM | 50:50 | 16.3 | 5.3 | 38.8 | 20.5 | 3.6 | 102.1 | 777.0 |
|  | 25:75 | 20.9 | 5.4 | 39.3 | 29.6 | 4.1 | 108.4 | 801.0 |
|  | 75:25 | 15.8 | 4.9 | 40.6 | 32.8 | 3.3 | 94.0 | 703.4 |
| WPNG+WC(2):SM | 50:50 | 19.1 | 5.3 | 36.0 | 36.3 | 4.1 | 102.0 | 754.7 |
|  | 25:75 | 15.5 | 4.8 | 38.8 | 32.3 | 3.7 | 106.6 | 789.4 |
| **LSD (α=0.05)** | | **3.2** | **0.8** | **7.1** | **9.1** | **n.s.** | **26.7** | **199.4** |

Table S5. The interaction of horticulture media and willow substrate:peat proportion on horticulture media chemical composition after tomato transplant production

| Horticulture  media | Willow substrate:peat proportion | pH | mS/cm | Nitrate | P | K | Mg | Ca |
| --- | --- | --- | --- | --- | --- | --- | --- | --- |
|  |  |  |  | mg ⋅ dm^-3^ | | | | |
|  | 75:25 | 6.89 | 0.51 | 7.5 | 93 | 250 | 59 | 525 |
| WC:SM | 50:50 | 6.88 | 0.57 | 3 | 77 | 213 | 52 | 870 |
|  | 25:75 | 6.31 | 0.72 | 4.2 | 55 | 110 | 56 | 1100 |
|  | 75:25 | 5.93 | 7.77 | 1800 | 131 | 300 | 140 | 870 |
| WN:SM | 50:50 | 5.95 | 6.58 | 1580 | 106 | 290 | 106 | 1010 |
|  | 25:75 | 6.15 | 3.31 | 580 | 67 | 155 | 82 | 1200 |
|  | 75:25 | 7.17 | 0.48 | 1 | 99 | 238 | 56 | 663 |
| WPG:SM | 50:50 | 7.15 | 0.51 | 1 | 47 | 150 | 48 | 870 |
|  | 25:75 | 7.1 | 0.71 | 5.8 | 68 | 100 | 62 | 1250 |
|  | 75:25 | 5.87 | 5.03 | 1280 | 109 | 275 | 64 | 1050 |
| WPGN:SM | 50:50 | 5.77 | 4.56 | 955 | 73 | 175 | 130 | 1230 |
|  | 25:75 | 6.03 | 2.45 | 642 | 62 | 50 | 88 | 1200 |
|  | 75:25 | 6.45 | 0.43 | 8.5 | 78 | 42 | 59 | 663 |
| WPNG+WC(1):SM | 50:50 | 6.38 | 0.39 | 6.9 | 55 | 45 | 62 | 870 |
|  | 25:75 | 6.48 | 0.62 | 12 | 66 | 30 | 68 | 1370 |
|  | 75:25 | 6.21 | 1.05 | 201 | 58 | 43 | 79 | 750 |
| WPNG+WC(2):SM | 50:50 | 6.27 | 0.89 | 150 | 46 | 32 | 72 | 1050 |
|  | 25:75 | 6.35 | 0.86 | 74 | 58 | 40 | 70 | 1450 |

Table S6. The interaction of horticulture media and willow substrate:peat proportion on cucumber plants parameters. One week from sowing

| Horticulture  media | Willow substrate:peat proportion | Plant height  (cm) | Leaf span  (cm) | Number of leaf | Plant conditions* |
| --- | --- | --- | --- | --- | --- |
|  | 75:25 | 5.0 | 10.0 | 1.0 | - |
| WC:SM | 50:50 | 5.3 | 10.3 | 1.0 | - |
|  | 25:75 | 5.2 | 10.3 | 1.1 | - |
|  | 75:25 | 0.0 | 0.0 | 0.0 | - |
| WN:SM | 50:50 | 1.8 | 3.5 | 0.0 | - |
|  | 25:75 | 3.4 | 6.3 | 0.1 | - |
|  | 75:25 | 6.1 | 10.7 | 1.2 | - |
| WPG:SM | 50:50 | 6.2 | 10.8 | 1.0 | - |
|  | 25:75 | 5.2 | 10.2 | 1.0 | - |
|  | 75:25 | 3.7 | 8.2 | 0.8 | - |
| WPGN:SM | 50:50 | 2.9 | 5.4 | 0.2 | - |
|  | 25:75 | 3.1 | 6.6 | 0.6 | - |
|  | 75:25 | 4.7 | 9.4 | 1.1 | - |
| WPNG+WC(1):SM | 50:50 | 5.4 | 9.6 | 1.1 | - |
|  | 25:75 | 5.0 | 10.5 | 0.9 | - |
|  | 75:25 | 3.6 | 7.4 | 0.6 | - |
| WPNG+WC(2):SM | 50:50 | 4.4 | 8.6 | 0.9 | - |
|  | 25:75 | 4.3 | 9.3 | 1.0 | - |
| **LSD (α=0.05)** | | 5.0 | 10.0 | 1.0 | **-** |

*no identificated

Table S7. The interaction of horticulture media and willow substrate:peat proportion on cucumber plants parameters. Two weeks from sowing

| Horticulture  media | Willow substrate:peat proportion | Plant height  (cm) | Leaf span  (cm) | Number of leaf | Plant conditions |
| --- | --- | --- | --- | --- | --- |
|  | 75:25 | 8.1 | 11.8 | 2.0 | 9.0 |
| WC:SM | 50:50 | 10.2 | 13.3 | 2.2 | 9.0 |
|  | 25:75 | 9.4 | 13.1 | 2.1 | 9.0 |
|  | 75:25 | 0.0 | 0.0 | 0.0 | 1.0 |
| WN:SM | 50:50 | 3.1 | 6.0 | 0.5 | 5.0 |
|  | 25:75 | 5.0 | 8.6 | 1.2 | 5.0 |
|  | 75:25 | 8.9 | 11.7 | 1.1 | 8.0 |
| WPG:SM | 50:50 | 10.6 | 13.3 | 2.0 | 9.0 |
|  | 25:75 | 9.3 | 13.6 | 2.2 | 9.0 |
|  | 75:25 | 6.5 | 10.6 | 1.9 | 8.0 |
| WPGN:SM | 50:50 | 3.8 | 7.0 | 1.2 | 5.0 |
|  | 25:75 | 5.5 | 9.0 | 1.5 | 5.0 |
|  | 75:25 | 8.4 | 12.9 | 2.0 | 9.0 |
| WPNG+WC(1):SM | 50:50 | 8.8 | 13.1 | 2.1 | 9.0 |
|  | 25:75 | 8.7 | 13.1 | 2.2 | 9.0 |
|  | 75:25 | 6.0 | 10.0 | 1.9 | 5.0 |
| WPNG+WC(2):SM | 50:50 | 7.5 | 11.5 | 1.9 | 8.0 |
|  | 25:75 | 8.5 | 12.5 | 2.0 | 8.0 |
| **LSD (α=0.05)** | | 1.2 | 1.6 | 0.3 | n.a. |

n.a. – no analyse

Table S8. The interaction of horticulture media and willow substrate:peat proportion on cucumber plants parameters. Three weeks from sowing

| Horticulture  media | Willow substrate:peat proportion | Plant height  (cm) | Leaf span  (cm) | Number of leaf | Plant conditions |
| --- | --- | --- | --- | --- | --- |
|  | 75:25 | 12.6 | 13.8 | 3.1 | 6.0 |
| WC:SM | 50:50 | 16.8 | 18.2 | 3.9 | 8.0 |
|  | 25:75 | 16.3 | 22.7 | 4.5 | 8.0 |
|  | 75:25 | 1.2 | 1.7 | 0.5 | 1.8 |
| WN:SM | 50:50 | 6.6 | 8.4 | 2.6 | 5.0 |
|  | 25:75 | 10.1 | 15.7 | 4.0 | 6.0 |
|  | 75:25 | 14.0 | 16.5 | 3.5 | 6.0 |
| WPG:SM | 50:50 | 16.6 | 20.6 | 3.8 | 8.0 |
|  | 25:75 | 16.4 | 21.2 | 4.4 | 9.0 |
|  | 75:25 | 9.9 | 14.3 | 3.4 | 6.0 |
| WPGN:SM | 50:50 | 7.8 | 10.0 | 2.2 | 6.0 |
|  | 25:75 | 13.3 | 18.9 | 4.2 | 6.0 |
|  | 75:25 | 16.2 | 22.6 | 4.4 | 9.0 |
| WPNG+WC(1):SM | 50:50 | 18.0 | 21.4 | 4.4 | 9.0 |
|  | 25:75 | 15.5 | 20.1 | 4.0 | 8.0 |
|  | 75:25 | 10.2 | 14.8 | 3.7 | 8.0 |
| WPNG+WC(2):SM | 50:50 | 11.3 | 17.8 | 3.9 | 8.0 |
|  | 25:75 | 13.3 | 18.5 | 4.0 | 8.0 |
| **LSD (α=0.05)** | | **1.9** | **2.7** | **0.6** | **0.2** |

Table S4. The interaction of horticulture media and willow substrate:peat proportion on tomato on tomato transplant parameters

Table S9. The interaction of horticulture media and willow substrate:peat proportion on tomato on cucumber transplant parameters

| Horticulture  media | Willow substrate:peat proportion | Transplant mass (g) | Steam diameter (mm) | Stocky plant index | SPAD | Leaf weight (g) | Leaf area (cm^2^) | Leaf area per plant (cm^2^) |
| --- | --- | --- | --- | --- | --- | --- | --- | --- |
|  | 75:25 | 8.6 | 5.0 | 30.2 | 12.4 | 4.1 | 132.7 | 420.5 |
| WC:SM | 50:50 | 12.1 | 6.8 | 25.0 | 16.0 | 6.6 | 223.6 | 879.0 |
|  | 25:75 | 19.3 | 7.3 | 22.6 | 24.1 | 9.0 | 319.0 | 1436.6 |
|  | 75:25 | 0.0 | 0.0 | 0.0 | 0.0 | 0.0 | 0.0 | 0.0 |
| WN:SM | 50:50 | 2.8 | 3.2 | 20.0 | 59.6 | 1.9 | 61.8 | 163.1 |
|  | 25:75 | 7.6 | 4.8 | 21.3 | 46.3 | 4.4 | 142.5 | 569.5 |
|  | 75:25 | 11.6 | 5.7 | 25.2 | 16.3 | 7.8 | 209.1 | 726.0 |
| WPG:SM | 50:50 | 13.0 | 5.8 | 28.8 | 17.8 | 5.1 | 205.5 | 779.3 |
|  | 25:75 | 15.4 | 6.5 | 25.4 | 34.7 | 6.3 | 221.4 | 988.3 |
|  | 75:25 | 4.8 | 4.6 | 21.6 | 36.2 | 2.6 | 88.2 | 300.1 |
| WPGN:SM | 50:50 | 3.6 | 3.9 | 20.7 | 38.4 | 2.6 | 80.1 | 179.2 |
|  | 25:75 | 12.2 | 6.2 | 21.8 | 44.1 | 4.7 | 169.0 | 703.5 |
|  | 75:25 | 17.2 | 6.5 | 25.1 | 25.4 | 8.1 | 290.7 | 1283.9 |
| WPNG+WC(1):SM | 50:50 | 18.4 | 6.8 | 26.6 | 29.8 | 8.6 | 294.1 | 1291.5 |
|  | 25:75 | 16.4 | 6.7 | 23.6 | 29.5 | 7.7 | 220.2 | 899.0 |
|  | 75:25 | 7.0 | 4.9 | 21.4 | 39.1 | 3.7 | 122.8 | 454.5 |
| WPNG+WC(2):SM | 50:50 | 9.3 | 5.2 | 21.9 | 35.5 | 4.4 | 142.9 | 559.3 |
|  | 25:75 | 11.4 | 5.4 | 24.6 | 32.3 | 4.6 | 162.3 | 647.1 |
| **LSD (α=0.05)** | | **n.s.** | **1.0** | **6.5** | **9.5** | **2.5** | **73.2** | **325.4** |

Table S10. The interaction of horticulture media and willow substrate:peat proportion on horticulture media chemical composition after cucumber transplant production

| Horticulture  media | Willow substrate:peat proportion | pH | mS/cm | Nitrate | P | K | Mg | Ca |
| --- | --- | --- | --- | --- | --- | --- | --- | --- |
|  |  |  |  | mg ⋅ dm^-3^ | | | | |
|  | 75:25 | 6.62 | 0.45 | 3.6 | 143 | 138 | 58 | 530 |
| WC:SM | 50:50 | 6.74 | 0.68 | 3.6 | 115 | 125 | 60 | 750 |
|  | 25:75 | 7.09 | 0.67 | 4.6 | 139 | 100 | 58 | 775 |
|  | 75:25 | 5.81 | 5.25 | 2530 | 146 | 475 | 232 | 1400 |
| WN:SM | 50:50 | 6.01 | 4.63 | 659 | 111 | 225 | 120 | 925 |
|  | 25:75 | 5.71 | 3.98 | 698 | 79 | 138 | 110 | 1050 |
|  | 75:25 | 7.13 | 0.44 | 2.6 | 92 | 87 | 58 | 338 |
| WPG:SM | 50:50 | 7.02 | 0.54 | 6.4 | 77 | 50 | 54 | 775 |
|  | 25:75 | 7.01 | 0.58 | 6.6 | 76 | 25 | 60 | 875 |
|  | 75:25 | 5.49 | 2.67 | 540 | 116 | 105 | 90 | 825 |
| WPGN:SM | 50:50 | 5.16 | 4.55 | 820 | 106 | 205 | 122 | 830 |
|  | 25:75 | 5.4 | 2.96 | 510 | 92 | 75 | 85 | 725 |
|  | 75:25 | 6.83 | 0.32 | 3.3 | 84 | 25 | 38 | 420 |
| WPNG+WC(1):SM | 50:50 | 6.83 | 0.45 | 12 | 73 | 23 | 48 | 1800 |
|  | 25:75 | 6.62 | 0.77 | 18 | 54 | 35 | 42 | 636 |
|  | 75:25 | 5.66 | 2.14 | 270 | 84 | 102 | 70 | 500 |
| WPNG+WC(2):SM | 50:50 | 5.83 | 2.01 | 213 | 76 | 20 | 64 | 900 |
|  | 25:75 | 6.11 | 1.26 | 247 | 65 | 50 | 56 | 625 |
